# Identification and In Silico Characterization of a Genetically Distinct Avian Rotavirus D Capsid Gene, *VP7*

**DOI:** 10.3390/pathogens7020037

**Published:** 2018-04-04

**Authors:** Pallavi Deol, Jobin Jose Kattoor, Shubhankar Sircar, Munish Batra, Kuldeep Dhama, Yashpal Singh Malik

**Affiliations:** 1 Division of Biological Standardization, ICAR-Indian Veterinary Research Institute, Izatnagar, Bareilly 243 122, Uttar Pradesh, India; pallavi.deol@gmail.com (P.D.); jobinjkattoor@gmail.com (J.J.K.); shubhankar.sircar@gmail.com (S.S.); 2 Department of Veterinary Pathology, G.B. Pant University of Agriculture and Technology, Pantnagar 263145, Uttarakhand, India; munishbatra74@rediffmail.com; 3 Division of Pathology, ICAR-Indian Veterinary Research Institute, Izatnagar, Bareilly 243 122, Uttar Pradesh, India; kdhama@rediffmail.com

**Keywords:** avian rotaviruses, rotavirus D, VP7 gene, phylogenetic analysis, domain identification

## Abstract

Rotavirus D (RV-D) is gaining importance as a cause of gastroenteritis and runting and stunting syndrome (RSS) in poultry. To date, information is scarce on the molecular analysis of RV-D isolates worldwide. In this study, the VP7 gene, a major constituent of outer capsid structural protein, from a RV-D isolate (UKD48) obtained from Uttarakhand state was analyzed. Phylogenetically, the RV-D isolate was found to be closely related to a South Korean strain, and the nucleotide percent identity varied from 80.4–84.2% with other RV-D strains available globally. Furthermore, domain investigation within 21 aligned amino acid sequences of the VP7 gene affirmed that this gene has several domains: a conserved glycosylation site (N–I–T) having an important role in protein folding; a N-terminal signal peptide (“ITG”) for endoplasmic reticulum retention; and two hydrophobic sites for elucidating transmembrane portions, antigenic structures, and so forth. The findings suggest that the VP7 gene of the Indian RV-D isolate is genetically distinct from those of other avian RV-Ds. Although biological evidence is still needed to prove the functional characteristics of these domains in outer capsid structural proteins, the present study adds new knowledge and derives the need for further investigation.

## 1. Introduction

Rotaviruses (RVs) have been identified as one of the main etiological agents of diarrhea and enteritis in mammals, including humans and avian species [1,2]. Belonging to the family *Reoviridae*, the RVs that infect birds, that is, avian rotaviruses (AvRVs), include A, D, F, and G species, which are classified on the basis of the antigenic properties of the VP6 protein. Among the four AvRVs, RV-A and RV-D exemplify high shedding frequencies. Apart from causing diarrhea, RV-D plays a key role in causing runting and stunting syndrome [3]. The RV genome is comprised of 11 linear segments of double-stranded RNA, and encodes six structural (VP1–VP4, VP6, and VP7) and five or six non-structural proteins (NSP1–NSP5/6) [4]. VP7 is a glycoprotein eliciting neutralizing antibodies and is crucial for defining the G type among RVs [5]. Hitherto, studies have proven the importance of glycosylation and hydrophobic/hydrophilic domains in protein folding, and the presence of the N-terminal signal peptide “ITG” in retaining VP7 in the endoplasmic reticulum membrane. This study highlights the genetic relatedness of the *VP7* gene of an RV-D isolate (UKD48) to other RV-D strains, N-glycosylation sites, distribution of hydrophobic/hydrophilic domains, and defines N-terminal signal peptide domains within the target gene sequences.

## 2. Materials and Methods

### 2.1. Sampling and Amplification of RV-D Capsid Gene

A sample (intestinal scraping) was collected from a young layer chick (UKD48) during postmortem from Uttarakhand state (India), and was subjected for the RV-D confirmation by using *VP6* gene-based RT-PCR [6]. Subsequently, the sample was used for *VP7* gene analysis with amplification of the complete coding sequence of the *VP7* gene using RT-PCR. Primers were designed (forward: 5′ AGGCATCTTAACCATATAGGAG 3′ and reverse: 5′ ATATCATAACTATTGGCGAACC 3′; nucleotide positions in reference strain GU733451: forward 10–31 and reverse 982–1003) to amplify a fragment of the *VP7* gene by identifying the consensus regions based on the nucleotide sequence alignment of *VP7* gene sequences obtained from the NCBI database. The optimized PCR conditions include an initial cycle of 94 °C for 3 min, followed by 35 cycles of 94 °C for 30 s, 48 °C for 45 s, 72 °C for 1 min, and a final extension cycle of 68 °C for 1 min using DreamTaq PCR master mix (Thermo Scientific, Waltham, MA, USA).

### 2.2. Cloning, Sequencing and Sequence Analysis

PCR-amplified product was resolved on 1.5% agarose gel, cloned into the pGEM-T vector system (Promega, Madison, WI, USA), and transformed. Plasmid was isolated using GeneJET Plasmid Miniprep Kit (Fermentas, Waltham, MD, USA), and positive recombinant clones were sequenced by SciGenom labs (Cochin, Kerala, India). Twenty sequences for the *VP7* gene of RV-D available in the NCBI database were retrieved. The CLUSTALW program of the version 6 of MEGA software package was used for the pairwise and multiple sequence alignment of retrieved sequences along with the present study sequence (UKD48) [7]. Open reading frames (ORFs) were identified and amino acid sequences were deduced using MEGA6. Aligned nucleotide and amino acid sequences were used to obtain the percentage identity against all the retrieved sequences using the MegAlign program of the DNAStar software package.

### 2.3. Phyloanalysis and In Silico Motifs/Domain Predictions of Capsid Gene VP7

A dendrogram was constructed using MEGA6 (v6.05) using the maximum likelihood method with the Tamura 3+G+I model for the *VP7* gene [7]. For the statistical reliability of dendrogram reconstruction, a bootstrap support value was set at 1000. Rotavirus-specific signature sequences and functionally important protein motifs were traced in the nucleotide sequences and amino acid sequences, respectively. TMHMM and Phyre2 servers were used for predicting the probability of each protein being located on the membrane surface, based on the hidden Markov model (http://www.cbs.dtu.dk/services/TMHMM/) [8]. To infer the protein structure and function of the deduced amino acid sequence, the Phyre2 protein fold recognition server was used (http://www.sbg.bio.ic.ac.uk/phyre2/) [9].

## 3. Results and Discussion

**Figure 1:**
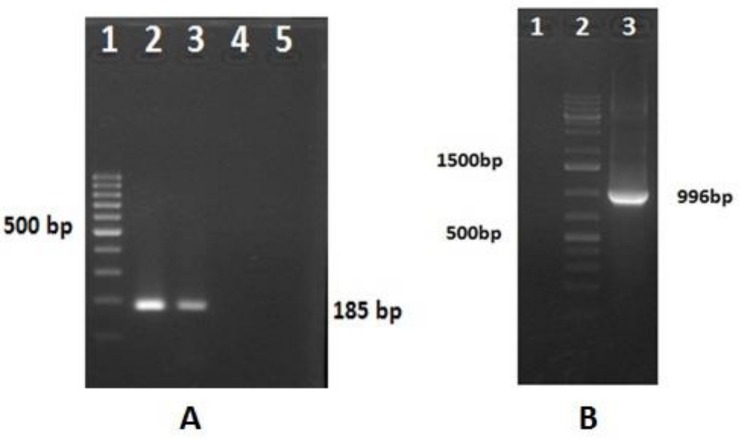
RT-PCR-amplified products of RV-D. (**A**) Lane 1: 100-bp DNA ladder (Thermo Scientific); lane 2: 185-bp amplicon of the *VP6* gene of RV-D; lane 3: positive-RVD insert plasmid as control; lane 4: non-template control; lane 5: empty well. (**B**) Lane 1: non-template control; lane 2: 1 kb plus DNA ladder (Thermo Scientific); lane 3: 996-bp amplicon of the *VP7* gene of RV-D.

The sample from the intestinal scraping collected from a young layer chick (UKD48) exhibiting signs of severe enteritis was confirmed for the presence of RV-D using *VP6* gene-based RT-PCR, yielding an expected amplicon of 185 bp (Figure 1A). Subsequently, the sample was used for the amplification of the complete coding sequence of the outer capsid protein gene *VP7*, using RT-PCR (Figure 1B). In the *VP7* gene-specific RT-PCR for RV-D, an amplicon of 996 bp corresponding to nucleotide positions 10–1005 in the reference strain (GU733451) was amplified (Figure 1B).

**Figure 2:**
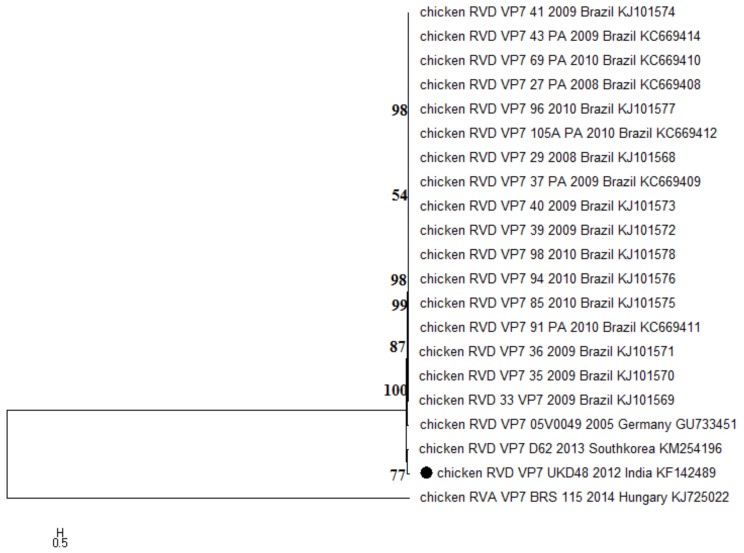
Maximum likelihood phylogenetic reconstruction of rotavirus D (UKD48 strain), based on the 996-bp complete coding sequence of the *VP7* gene. The Tamura three-parameter substitution model with the evolutionary invariable method (T3+G+I) was identified using the “find best DNA/protein model tool available” in MEGA6, which was confirmed with the FindModel online tool. Only >50% bootstrap values are shown. Numbers on branches indicate the percentages of bootstrap support from 1000 replicates. The analysis involved 21 nucleotide sequences, including one study strain (UKD48), denoted by the solid dot, and one chicken RV-A sequence as an outgroup. The scale bar indicates nucleotide substitutions per site.

Evolutionary analysis of the UKD48 strain (accession no. KF142489) of RV-D revealed that the sequences fell into two distinct major clusters, wherein the present study sequence was found neighboring the South Korean RV-D strain D62 (KM254196) (Figure 2). Notably, Asian RV-D sequences formed an entirely separate clade from Brazilian sequences. The percent identity of the UKD48 strain varied from 80.4 to 84.2% and 84.3 to 90.4% at the nucleotide and amino acid levels, respectively. As of now, only 20 RV-D *VP7* gene sequences are available in the public domain, the majority of which are from Brazil. Thus, there is a need to exemplify the work on the genetic characterization of RV-D isolates from different parts of the world for understanding the virus’ evolution mechanisms.

**Figure 3:**
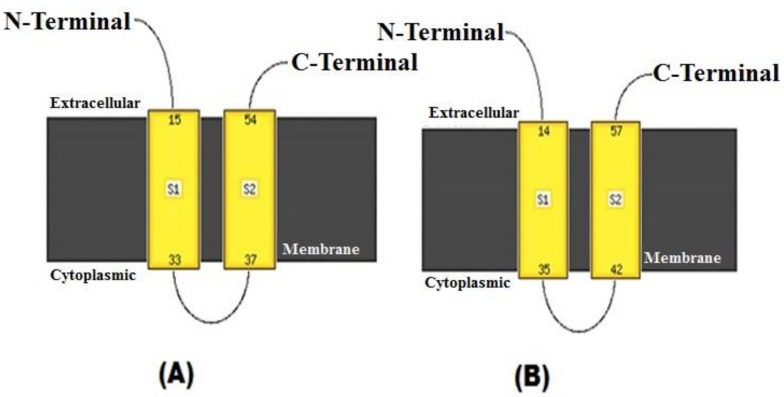
Analysis of hydrophobic domains in rotavirus D and rotavirus A *VP7* gene sequences. Depiction of the yellow regions shows the hydrophobic domains as S1 and S2. (**A**) S1: 15–33 and S2: 37–54 of RV-D, and (**B**) S1: 14–35 and S2: 42–57 of avian RV-A (GU733451)) within the *VP7* gene. The domains were predicted using Phyre2 [9].

Apart from phylogenetic findings, functionally important domains in the aligned amino acid sequences were analyzed. It is interesting to note here that one N-glycosylation site, N–X–S/T (where X can be any amino acid except proline), was found conserved in the form of N–I–T (243–245) in all the RV-D sequences, which is in contrast with the earlier studies done by Trojnar and coworkers, where no N-glycosylation site was evident [10]. Glycosylation sites are important for protein folding [11], and may lead to varied polypeptide patterns among different strains. Even though, hitherto reports confirm that outer capsid VP7 protein is capable of generating neutralization antibodies in all the host species and the RV-A VP7 protein possess few neutralization domains, namely A (87–101), B (141–151), and C (208–224) [12,13]; as such, no conserved neutralization domains in the aligned amino acid sequences of UKD48 and other RV-D isolates were noticed. Furthermore, since VP7 is an endoplasmic reticulum membrane-associated glycoprotein in RVs, a N-terminal signal peptide, “ITG”, which is specifically necessary for the retention of VP7 in the endoplasmic reticulum in the case of RV-A (I9, T10, and G11) [14], was present at positions 119, 120, and 121 of the RV-D VP7 protein and was conserved. However, whether “ITG” at this position in RV-D serves the same function as described for RV-A needs to be validated using site-specific mutagenesis and transfection techniques. Moreover, like two hydrophobic regions in the VP7 protein of RV-A (14–35, 42–57), two stretches of hydrophobic regions were found in RV-D (15–33, 37–54) (Figure 3). The distribution of hydrophobic/hydrophilic domains provides insights into the overall folding pattern of the protein, which eventually helps in elucidating the transmembrane regions, antigenic structures, and so forth.

The findings of the current study, based on the low sequence identity and distinct clade in the phylotree, suggest that the Indian RV-D isolate (UKD48) is genetically divergent from other avian RV-Ds. Furthermore, the *VP7* gene contains several important domains as seen in the case of mammalian RV-A, including one N-glycosylation site which plays a role in protein folding and cellular attachment. However, further biological studies are necessary to support this data.
